# A Case of Apoplexia Hydrocephalica, with Remarks

**Published:** 1801-02

**Authors:** T. Garnett

**Affiliations:** Professor of Natural Philosophy and Chemistry in the Royal Institution of Great Britain; Honorary Member of the Board of Agriculture, &c. &c.


### *A Case of Apoplexia Hydrocephalica, with Remarks.*

By T. Garnett, M. D.

*Professor of Natural Philosophy and Chemistry in the Royal Institution of Great Britain; Honorary Member of the Board of Agriculture, &c. &c.*

On the 19th of November in the evening, I was requested to visit Miss H. aged 16, whom I found in a comatose state; her breathing was rather strong and unequal, and when roused by considerable force she did not open her eyes, but put her hand upon her forehead, and cried out in a most piteous manner, "O my head!" Her pulse was 96, and rather full, and the catamenia, which had been regular for near two years, had appeared the preceding week. The patient when in health was of a very lively disposition and remarkably intelligent. About a year before, she had complained for a few days of pain in her head, which was removed by gentle purgatives.

For two days before I visited her, she had complained of considerable pain in the forehead, extending on each side to the temples. When I first saw her, I could with great difficulty get her to speak; but when at length she was sufficiently roused, she said the pain was so violent that she thought her skull was separating, and immediately relapsed into a state of insensibility. The pain had become more violent about two hours before, previous to which some symptoms of hysteria had appeared, which were succeeded by violent sickness and vomiting.

With a view of alleviating the present symptoms, I ordered her the following draught:

℞. Tinct. opii gutt. xxv.—Æther. vitriolic ʒj.—Aqu. fontan. ℥ß.—Syr. simp. ʒj. M.

And as there was some appearance of nervous irritability, I prescribed the following:

℞. Spir. vol. fœtid.—Tinct. valerianæ vol. Spt. lavend. comp. aa. ʒij.

Of this mixture a tea spoonful was directed to be taken every three hours in a little water.

Nov. 20th.—She had passed a very restless night; her sleep was disturbed; and at intervals she complained much of pain in her head, shooting downwards towards her left shoulder. She had been very sick in the night, and had thrown up the drops twice, but not the draught. Pulse 100, full, and somewhat irregular. She seemed always much inclined to sleep, and when roused, almost instantly closed her eyes: She appeared however to have considerable aversion to the light, and desired that the curtains of the bed might be close drawn, in order to exclude it; notwithstanding which, the pupils of her eyes were preternaturally dilated, and scarcely contracted on bringing a lighted candle near them. The pain in her head she said was very acute, and never left her for one instant. Her skin was very hot and dry; and as she had not had an evacuation of the bowels for two days, I directed a stimulant glyster, which brought away some hardened fæces.

As I had now reason to apprehend that I had to combat that terrible disease, the Apoplexia Hydrocephalica, I directed the application of a large blister between the shoulders, and one of the following pills to be taken twice a day:

℞. Calomel.—Pulv. digital. purpur. aa. gr. vj.—Extract. gentian. q. s. ut ft. Pil. vj.

I likewise ordered a drachm of *unguent. hydrarg. mit.* to be well rubbed on the nape of the neck and behind the ear, three times a day. I also directed the immediate application of three leeches to each temple.

21.—The leeches had drawn a considerable quantity of blood, and she thought her head rather easier for some hours; but the pain was now as bad as ever; the comatose disposition had increased, and in the night she had been delirious. The pupils of her eyes were much dilated, and did not contract on the approach of a candle. Her pulse was 76, irregular, and somewhat strong. She did not complain of pain any where excepting the head.

22.—Much the same as yesterday.

23.—Pain of the head very violent and incessant; pulse only 60, but full and regular. She frequently lay a long time without breathing, and then sighed deeply. The pupils were still dilated, and she had an evident strabismus, the axis of the right eye being directed towards the nose. As she continued to be costive, the glyster was ordered to be repeated.

24.—Pulse only 52, very regular, not quite so much stupor, but the pain of the head very violent. Strabismus and dilated pupils as before. Had voided a considerable quantity of high coloured urine during the night; she had also begun to spit a good deal, but did not complain of soreness of the mouth.

25.—Pulse 60, regular and full; less stupor; had slept none in the night, but sighed frequently; complained much of the pain of her head, which she said was more violent than ever, and extended towards the *occiput*. As the blister between the shoulders was healed, I directed a small one to be applied behind each ear. She had ejected upwards of a pint and a half of saliva, and voided a large quantity of urine. In order to check the salivation, which I was apprehensive might go to a greater length than I wished, I directed eight grains of *kali sulphuratum* to be taken taken every two hours in a little water. This, as I have elsewhere obferved,* is always found very efficacious in checking or putting a ftop to falivation. In 24, or at moft 48 hours after the firft exhibition of this remedy, the falivation becomes much abated. It has been reafonably fuppofed that mercury derives moft of its activity from being in the ftate of an oxid; for crude mercury poffeffes little or no power, and in the form of mercurial ointment, the mercury is evidently oxygenated by continued friction. On the decompofition of the water in which the medicine is given, by the kali fulphuratum, fulphurated hydrogen gas is produced, which will be conveyed into the blood, where the hydrogen unites with the oxygen of the mercurial oxid, and forms water, while the fulphur will convert the metal into an ethiops or fulphuret, which is very inert.

With a view of affording fome relief to the excruciating pain of the head, I directed a pill of opium to be taken at bed time.

26.—Pulfe 80, very full and regular. Had flept none, but been delirious the greateft part of the night; pain intolerable.—Strabifmus ftill remains, but the pupils contract a little on the approach of light. Salivation diminifhed. The quantity of urine not fo great, but ftill confiderable. The pills and mercurial ointment had been omitted fince yefterday. To alleviate the pain, the temples and forehead were rubbed with ether; but this proving ineffectual, three leeches were applied to each temple, and one of the following powders was directed to be taken every three hours in a little tea.

℞. Nitri purif. ʒij.—Sacchar. alb. ʒiij.—Pulv. coccinell. gr. viij. M. et divid. in part. xij.

27.—The pain of the head was relieved for fome hours after the application of the leeches, but had returned, though not quite fo violent as before. Pulfe 80, not quite fo full. No perceptible ftrabifmus, but the pupils continue dilated. The powders were ordered to be continued.

28.—Had flept a little, but ftill complained of great pain in the head. Pulfe 84, rather full. Salivation gone, urine natural. As fhe naufeated the nitrous powders, an effervefcing faline draught was directed to be taken every four hours.

30.—Pain of the head ftill bad, though not quite fo acute. Pulfe 80, not fo ftrong. Has had a ftool every day for the laft three days. The medicines continued.

December 1.—Pain of the head very bad, but flept a little laft

* Letter to Dr. Beddoes, publifhed in his Confiderations on the medicinal ufe and production of factitious Airs, p. 115. 2d edition.

laſt night. Pupils conſiderably dilated. Pulſe 96, of natural ſtrength, and regular.

The pills of calomel and digitalis were ordered to be repeated.

2.—Had two ſtools yeſterday. Slept a little during the night. Pain of the head and pulſe as yeſterday. The effervefcing draught being very grateful, was ordered to be continued as well as the pills.

4.—Pain leſs acute. Pulſe 96, and regular. Pupils not ſo much dilated, and contract more on the approach of light. Sat up three hours yeſterday. The bliſters diſcharge well. The pills were directed to be continued, and the friction with mercurial ointment to be reſumed.

6.—Pain much leſs acute; has ſat up ſeveral hours. Pulſe natural, pupils not dilated; ſlept well. Remedies to be continued.

8.—Pain gone. Complains of nothing but weakneſs. The pills and friction to be omitted.

12.—No complaint but that of being rather coſtive.

℞. Maſs. pil. Rufi ʒjſs.—Calomel. ℈j.—Syr. ſimp. q. s. ut ft. Pil. xx.—Sumat j. vel ij. pro re nata.

31.—No return of the complaints.

During the worſt period of her illneſs it may be obſerved, that ſhe never aſked for any thing; but when food of any kind was offered her, ſhe took it very eagerly. She was not afflicted with vomiting, except during a few of the firſt days.

### REMARKS.

From a view of the ſymptoms in the preceding caſe, there can be little heſitation in referring them to the diſeaſe which Dr. Whytt calls *Hydrocephalus internus*; but which Dr. Cullen, perhaps with more propriety, calls *Apoplexia hydrocephalica*, though I think this term not altogether unexceptionable, for reaſons which I ſhall afterwards mention.

All the ſymptoms enumerated by this accurate Noſologiſt, in his definition, were preſent in this caſe. "Apoplexia *(hydrocephalica),)* paulatim adoriens; infantes et impuberes, primum laſſitudine, febricula et dolore capitis, dein pulſu tardiore, pupillæ dilatatione, et ſomnolentia afficiens."*

The ancient phyſicians ſeem to have had but little knowledge of this diſeaſe, and it is even only within theſe few years that we have had any accurate accounts of it. Whether this diſeaſe is now more common than formerly, as appears to be the caſe with ſome others, I cannot determine, but am of opinion that it was generally miſtaken; and though in early medical writers, we

* Synopſis Noſologiæ Methodicæ, vol. II. p. 187.

we meet with defcriptions of fymptoms fimilar to thofe enumerated in the definition, they were thought in general to proceed from worms, and treated accordingly.

Dr. Whytt was the firft who publifhed any accurate defcription of this diforder, and of the appearances on diffection. Afterwards, in the London Medical Obfervations and Inquiries, vol. iv. we meet with an excellent Effay on this difeafe, by the late Dr. Fothergill.

Moft authors who have defcribed it, agree in their account of its fatality. Sennertus fays, " Si poft dolorem vehementem, magna fequatur pupillæ dilatatio, exigua fanationis fpes eft."* Dr. Fothergill alfo fays, " At the fame time I muft own to you, it is not in my power to fuggeft any probable means of curing the difeafe of which I treat; it has baffled all my attempts, both when confided in alone, and in confultation with the ableft of the Faculty. All I pretend to do is, to exhibit fuch an idea of this difeafe as may ferve to make it known when it occurs in practice, and to form fuch a prognoftic of its progrefs and event, as may juftify practitioners to themfelves and to the families in which fuch fatal occurrences may prefent themfelves."† Dr. Whytt freely owns that he has never been fo fortunate as to cure any one patient, whofe fymptoms denoted its exiftence with any degree of certainty.

Within thefe few years I have feen feveral inftances of this malady, and though I have only been completely fuccefsful in the prefent and two other cafes, yet there are now upon record feveral fuccefsful cafes; and I am in hopes that when we become better acquainted with the nature of the difeafe, the fuccefsful cafes will counterbalance thofe of a contrary defcription. Since the time of Dr. Fothergill, this difeafe has attracted the attention of feveral eminent phyficians, particularly of Drs. Percival, Haygarth, Dobfon, &c. who have all favoured the public with valuable obfervations on it. The beft account of this difeafe is however given us by Dr. C. W. Quin of Dublin, in " A Treatife on the Dropfy of the Brain." He has given a number of valuable and highly important fuggeftions with refpect to the treatment as well as the theory of this difeafe, and fo accurate a defcription of it, that practitioners will now be fcarcely at a lofs in diftinguifhing it. A general outline of his opinions on this fubject was given in his Inaugural Differtation, publifhed at Edinburgh in the year 1779. Since that period, he fays, that neither facts nor arguments have occurred of fufficient force

* Lib. I. Part 3, Sect. 2. Chap. 26. Pract.

† Med. Obf. and Inquiries, Vol. iv.

force to weaken the conviction of their truth; on the contrary, his theory can now appear supported by an added strength of evidence, which minute attention to the subject has enabled him to draw from books, and observation in a variety of cases.

Within these last six years I have seen several instances of this disease and though only in the cases I have mentioned; have I been able to effect a cure, yet in two others the symptoms were so much alleviated by what is generally called the antiphlogistic plan, conjoined with mercurials and digitalis, that I had great hopes of their recovery. But the misfortune is, that a physician is generally consulted after the inflammatory stage is past, in which alone any good can be done.

Since Dr. Whytt favoured the world with his excellent history of this disease, I think it has been nearly a general opinion that children are almost the only subjects liable to its attacks, and chiefly those between the fourth and tenth years of their age; and I am of opinion that the symptoms which indicate the presence of this disease, seldom show themselves in an advanced period of life, but are much oftener met with in infants; yet from the observations of Dr. Quin and my own experience, this disease does not by any means appear to be confined within the limits just mentioned. The subject of this case was sixteen, I had one patient who was thirty years of age, and another thirteen. Dr. Quin relates some cases of patients forty years of age, though such instances are certainly not very common in the usual course of practice.

The proximate cause of this disease has hitherto been involved in considerable obscurity, and consequently the practice must have been guided by vague and uncertain principles. A due consideration of the various circumstances which present themselves in this disease, together with the appearances on dissection, would induce us to suspect, on very probable grounds, that the disease depends upon, and is generally, if not always, accompanied by a plethoric state of the vessels of the brain, occasioning a considerable degree of inflammation, and generally, (though not always,) producing an extravasation of watery fluid before death. As proofs of the existence of this inflamed state of the vessels of the brain, I beg leave to offer the following circumstances. In all cases where blood has been drawn, it exhibits the same appearance as in cases which are undoubtedly inflammatory. In some of the cases which fell under my observation, the blood had this appearance in as great a degree as I have ever observed in pleurisy. The acuteness of the pain and fulness of the pulse likewise countenance this supposition; but what amounts almost to a demonstration, is the aggravation of the symptoms from the exhibition hibition of ftimulants; and the relief which in the firft ftage of the difeafe, always attends the antiphlogiftic or debilitating plan; both of which were feen in the cafe juft related. The appearances on diffection likewife confirm this. Dr. Quin mentions the appearances which prefented themfelves in the brain of a young woman who died at Edinburgh in the year 1777, and that of a boy attended by his father fome years ago in Dublin.* In both of thefe cafes, the blood veffels of the brain were fo unufually diftended, that the whole cerebrum and cerebellum refembled an anatomical preparation in which the utmoft force of injection had been employed. What is very remarkable in thefe cafes, is, that though the fymptoms preceding death were fo unequivocal, yet in both cafes, to the aftonifhment of thofe who were prefent, and who expected to find a confiderable quantity of water in the cranium, none could be difcovered.

In four other cafes mentioned by Dr. Quin, where the quantity of effufed fluid found in the ventricles was confi-derable, there were figns fo vifible of an increafed flow of blood to the brain, that in all of them the veffels were remark-ably turgid, and in moft of them a great degree of inflam-mation had actually taken place.

The accurate Morgagni alfo relates feveral inftances in which the fymptoms of this difeafe had been prefent, and where, upon diffection, evident marks of morbid accumulation, and of an inflammatory ftate of the veffels of the brain, had appeared.†

All thefe circumftances ftrongly tend to confirm the fup-pofition that the fymptoms, particularly thofe which appear in the firft ftage of *Apoplexia Hydrocephalica*, depend upon a plethoric and inflammatory ftate of the veffels of the brain. And though in many cafes, upon diffection, a quantity of fluid has been found in the ventricles of the brain, yet may not this be looked upon as a confequence of the difeafe, rather than as the caufe of it? And is it not probable that this watery effufion does not take place till the veffels have been oppreffed for fome time by the exceffive congeftion of blood, and in confequence of this, and the violent excitement, have loft their tone; whence the aqueous fluids are difcharged in con-fiderable quantities into the adjacent cavities, while the ab-forbents, probably weakened by the fame caufe, are unable to abforb

* Quin on Dropfy of the Brain, p. 50.

† Vide Morgagni de Caufis et Sedibus Morborum, Epift. i. Art. 2, Epift. vi. Art. 6, and Epift. i. Art. 14.

abforb the fluid as faft as it is effufed? It appears to me even doubtful whether the fymptoms which generally fhow themfelves in this difeafe, depend upon the effufed fluid. Many of them undoubtedly do not; and in the two cafes related by Dr. Quin, "all the fymptoms that generally attend this difeafe were prefent, and yet upon diffection, to the aftonifhment of the fpectators, who were prepared to find a great redundancy of water within the cranium, none could be difcovered in any part of the brain." Surely, the name *hydrocephalus* is not applicable to fuch cafes.

In every cafe therefore, it would be proper in the firft ftage, if no fymptoms particularly forbid, to ufe the debilitating plan to a confiderable extent. I have made ufe of it in three cafes which have been cured, and have feen others much relieved by it; at the fame time however it muft be noticed, that the other remedies foon to be mentioned, were adminiftered, for the difeafe generally runs on fo rapidly to its fatal period, that I think it would not be juftifiable to neglect any of the means likely to arreft its progrefs.

In ftrong fubjects, efpecially thofe who have attained the age of puberty, general bleeding may be ufed; but I fhould rather prefer local bleeding, becaufe eventually a great degree of debility comes on; and it feems probable that the accumulation in the veffels of the head may be moft effectually relieved by evacuations made as near the part as poffible. I am convinced, that there often exifts, not only in this difeafe but in fome others of the inflammatory kind, a local inflammation without much general fthenic diathefis; and when the veffels have taken on an inflammatory action in any part, general bleeding may be employed to fuch an extent as to weaken the fyftem very much, without confiderably abating the increafed action, while local bleedings, efpecially after one general evacuation, often produce a fpeedy folution of the inflammation. This is the cafe particularly with the acute rheumatifm.

In fome cafes it might be advifable to open the jugular vein, or even the temporal artery, but I have never yet feen either of thefe operations performed in this difeafe. As in almoft all cafes, coftivenefs takes place, and aggravates the fymptoms, it will be right to keep the bowels open by means of purgatives exhibited by the mouth, or ftimulant glyfters. In all cafes I have had recourfe to blifters, and I think with advantage. Where it has not been objected to, I have applied a blifter to the whole head; in other cafes I have directed them to be applied behind the ears, or between the fhoulders.

The greateft chance of cure is during the inflammatory ftage, and by means of the antiphlogiftic regimen carried to its its fulleſt extent. Whether in this ſtage much is to be expected from mercurials, I cannot ſay. They are highly ſpoken of by Drs. Percival and Dobſon in the Medical Commentaries; and on account of this reſpectable authority, I have always preſcribed them, as ſoon as the nature of the diſeaſe could be aſcertained. That mercurials have been employed with ſucceſs appears likewiſe from the teſtimony of Dr. Moſeley.* Alſo from that of Drs. Hunter and Haygarth.† Is it not probable that this remedy may produce its good effects by exciting the action of the abſorbents, and thus enabling them to take up the extravaſated fluid? and would they have produced any good effects in the two caſes related by Dr. Quin, where there was no watery effuſion? The digitalis being one of the moſt powerful diuretic remedies with which we are acquainted, is a very proper remedy in this diſeaſe; and I think I have found that its diuretic effects are much more certain and powerful, when conjoined with mercury. The digitalis likewiſe claims attention on other grounds. It ſeems to poſſeſs the power of diminiſhing the action of the arterial ſyſtem in a very remarkable degree, and is therefore indicated in the inflammatory ſtage.

From the attention which phyſicians have of late years paid to this diſeaſe, we ſhall I hope be ſhortly in poſſeſſion of ſufficient facts to eſtabliſh a rational theory of it, which will lead to a ſucceſsful plan of cure. Should theſe remarks contribute ever ſo little to the attainment of ſo deſirable an end, I ſhall be highly gratified.

THOMAS GARNETT.

---

### *To the Editors of the Medical and Phyſical Journal.*

GENTLEMEN,

BY enquiry and diſcovery, the condition of man is improved; and by the continued application of talent and induſtry to the art of healing, it is to be expected that much will be atchieved. The old fabrics of phyſic will undergo a cloſe examination; the rubbiſh will be removed, and the valuable materials only will be ſuffered to remain: the myſterious pe-

* London Medical Journal, Vol. vi. p. 113.

† Medical Obſervations and Inquiries, Vol. vi.

